# 87 A co-creative healthy lifestyle program for social vulnerable community dwelling older adults

**DOI:** 10.1093/eurpub/ckae114.004

**Published:** 2024-09-26

**Authors:** Pieterjan Verschelden, Julie Vanderlinden, Sabine Lambers, Eva Es

**Affiliations:** Odisee University Of Applied Sciences, Department Of Health Care, Brussels, Belgium; Odisee University Of Applied Sciences, Department Of Health Care, Brussels, Belgium; University of Brussels, Medical sciences (Pain in motion research group), Brussels, Belgium; Odisee University Of Applied Sciences, Department Of Health Care, Brussels, Belgium; Odisee University Of Applied Sciences, Department Of Health Care, Brussels, Belgium

## Abstract

**Purpose:**

Social vulnerable older adults tend to have a lower health literacy regarding healthy behaviours and they most often experience limited access to healthy lifestyle programs. Existing evidence-based lifestyle programs are not always accessible or are not tailor-made for social vulnerable older adults. The overarching purpose of the program is to introduce and empower social vulnerable older adults to healthy lifestyle behaviours.

**Description:**

Motivators, barriers and needs of social vulnerable older adults regarding healthy lifestyle behaviours were identified by means of a literature review and a needs assessment. Based on this assessment and literature, a healthy lifestyle program (AHAA) was developed in co-creation with a multidisciplinary team of researchers, older adults, local community centres and students in healthcare. A pilot version of the AHAA program was implemented in four local community centres in Flanders and Brussels. Three revision rounds, entailing a neighbourhood analysis and focus groups helped shape the final AHAA program. The AHAA program consisted of ten weekly sessions that were focused on topics such as physical activity and sedentary behaviour, fall prevention, healthy nutrition, stress and sleep, positive psychology, meaningful living and motivation.

The program was then implemented in six local community centres in Flanders and Brussels.

In order to make the program self-sustaining, staff and volunteers of the local community centres received training in order for them to be able to provide all AHAA sessions.

**Conclusions:**

The neighbourhood analysis facilitated a broader reach within these social vulnerable older adults but the recruitment of social vulnerable older adults required remains challenging. In terms of the social vulnerability of this target group, it is important to highlight the expertise of these older adults in the co-creation and throughout the development and implementation of the program by including them as experts in ageing.

According to the participants, the belongingness and ‘fun’ factor in AHAA turned out to be success factors for the adherence to the program. The participating local community centres experienced an increase in numbers of older adults that were attracted to their centre as well as an increase in their activities that are focused on healthy lifestyle behaviours.

